# Pressure-Induced Structural Effects in the Square Lattice (**sql**) Topology Coordination Network Sql-1-Co-NCS·4OX

**DOI:** 10.1021/acs.cgd.2c00982

**Published:** 2022-10-31

**Authors:** Ewa Patyk-Kaźmierczak, Michał Kaźmierczak, Shi-Qiang Wang, Michael J. Zaworotko

**Affiliations:** † Department of Materials Chemistry, Faculty of Chemistry, Adam Mickiewicz University in Poznań, Uniwersytetu Poznańskiego 8, 61-614Poznań, Poland; ‡ Department of Chemical Sciences and Bernal Institute, University of Limerick, Co. LimerickV94T9PX, Ireland

## Abstract

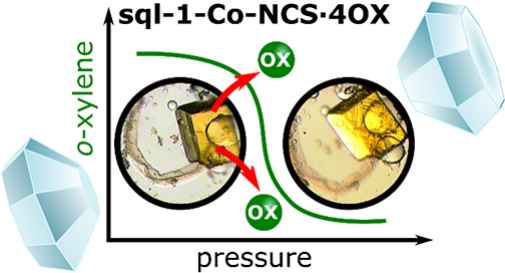

A high-pressure study of a switching coordination network of square lattice topology (**sql**) loaded with *o*-xylene (OX), [Co(4,4′-bipyridine)_2_(NCS)_2_]_*n*_·4nC_8_H_10_ (sql-1-Co-NCS·4OX), was conducted up to approximately 1 GPa to investigate pressure-induced structural changes. Previous reports revealed that sql-1-Co-NCS exhibits multiple phases thanks to its ability to switch between closed (nonporous) and several open (porous) phases in the presence of various gases, vapors, and liquids. Networks of such properties are of topical interest because they can offer high working capacity and improved recyclability for gas adsorption. The monoclinic crystal structure of sql-1-Co-NCS·4OX at 100 K was previously reported to show an increase in interlayer separation of more than 100% compared to the corresponding closed phase, sql-1-Co-NCS, when exposed to gases or vapors under ambient conditions. Herein, a tetragonal crystal form of sql-1-Co-NCS·4OX (space group *I*4/*mmm*, Phase I) that exists at 0.1 MPa/303 K is reported. Exposure of Phase I to high pressure using penetrable pressure transmitting media (OX and 1:1 vol MeOH/EtOH) did not result in further separation of the **sql** networks. Rather, compression of the crystals and release of adsorbed OX molecules occurred. These pressure-induced changes are discussed in terms of structural voids, framework conformation, and molecular packing of the **sql** layers. Although Phase I retained tetragonal symmetry throughout the investigated pressure range, the interlayer voids occupied by OX molecules were significantly reduced between 0.3 and 0.5 GPa; further compression above 0.5 GPa induced structural disorder. Additionally, analysis of the electron count present in the pores of sql-1-Co-NCS confirmed the multistep evacuation of OX molecules from the crystal, and two intermediate phases, I*a* and I*b*, differing in the OX loading level, are postulated.

## Abstract 2

Pressure-induced guest desorption, proceeding via two intermediate, partially emptied phases, is observed for crystals of a switching coordination network of square lattice topology loaded with *o*-xylene (sql-1-Co-NCS·4OX).

## 1 Introduction

Metal–organic frameworks (MOFs),^1,2^ or porous coordination polymers (PCPs),^3−5^ are a class of porous metal–organic materials (MOMs)^6,7^ that have been intensively researched in recent decades.^8,9^ Interest in such materials has been driven mainly by two factors: their amenability to crystal engineering due to their modular nature, which in turn enables systematic control over pore size and chemistry, and their potential utility for storage, separation, sensing, and catalysis.^10^ Such porous materials are also promising in the context of storage of gaseous fuels,^11^ as they can address problems associated with current storage technologies, i.e., low volumetric energy density.^12^ Whereas more than 100,000 MOFs have been reported, over 0.5 million are predicted.^13^ Unfortunately, most reported MOFs exhibit type-I isotherms^14,15^ characteristic of rigid microporous materials.^16^ For such materials, the working capacity cannot be as high as the saturation uptake since a significant amount of sorbate remains at the delivery pressure.^15^ Sorption applications are also mitigated by poor recyclability and/or hydrolytic instability.^17−19^ These issues, often associated with rigid 3D MOFs, can be overcome by layered coordination networks (CNs) that switch between closed (nonporous) and open (porous) phases and thereby exhibit ″S-shaped″ (type F-IV) isotherms, which enable a high working capacity.^15,20,21^ Additionally, the low mechanical strain in nonporous and porous phases can improve recyclability^15^ and increase uptake capacity.^22^

With respect to layered CNs that have been investigated in the context of gas sorption, square lattice (**sql**) topology CNs stand out. The first noninterpenetrated bipyridine-based **sql** CN was reported in 1994 by Fujita’s group.^23^ Such CNs are of the general formula [M(L)_2_(A)_2_]_*n*_ and are composed of octahedral metal nodes (M), linear ditopic linker ligands (L), and terminal axial ligands, usually simple inorganic anions (A). Several adsorption studies of **sql** CNs have been reported in the literature.^15,20,22,24−30^ Notably, the sorption behavior of ELM-11 [Cu(4,4′-bipyridine)_2_(BF_4_)_2_]_*n*_ revealed switching from closed to opened phases in the presence of CO_2_,^20,25,26^ O_2_,^24^ N_2_,^24,25^ CH_4_,^24,25^ C_2_H_2_,^27,28^ and *n*-butane.^26^ Another variant, [Co(4,4′-bipyridine)_2_(NCS)_2_]_*n*_ (sql-1-Co-NCS),^31,32^ not only showed switching upon exposure to CO_2_,^15^*o*-xylene (OX), *p*-xylene (PX), *m*-xylene (MX), and ethylbenzene (EB) but also efficiently separated C_8_ aromatics by means of physisorption.^22^ Furthermore, the interlayer separation (i.e., the distance between the adjacent **sql** networks) observed for sql-1-Co-NCS upon closed-to-open transformation after OX adsorption is the highest reported so far for the 4,4′-bipyridine-based **sql** CNs.^22^ The switching nature of sql-1-Co-NCS is impacted by external stimuli, as shown by the study of the framework loaded with OX and MX. Single crystals transformed into four-domain twins when the temperature was lowered from 298 to 100 K, an indication of a phase transition.^22^ This sensitivity to temperature could be an indicator that structural changes can also be induced by other stimuli, such as high pressure (≥0.1 GPa).^33^

Numerous studies have shown that extreme pressure can significantly affect the structure of MOFs,^34,35^ with the earliest high-pressure depositions in the MOF subset of the Cambridge Structural Database (CSD)^36^ dating back to 2006.^37^ Since then, the number of high-pressure CN structures deposited in the CSD has grown exponentially^38^ and now exceeds 750.^39^ High-pressure investigation of CNs allows us to assess their stability, fracture toughness, elasticity, stiffness, and hardness, factors affecting their possible applications.^40^ The susceptibility of the MOFs’ coordination environment and ligands to distortion may lead to more extreme structural transformations that occur at lower pressure than in zeolites.^34,41^ Moreover, for charge neutral MOFs, a pressure-induced guest exchange can be nondestructive.^42^ Additionally, previous reports have shown that high-pressure effects in MOFs can be relevant to their behavior under ambient conditions, for example, providing insight into their breathing/switching mechanism.^34,35,43^

The response of CNs to extreme pressure is strongly dependent on the type of the pressure transmitting medium (PTM).^44,45^ The size of PTM molecules with respect to the pore size of MOFs will determine whether the PTM can or cannot penetrate the framework. Therefore, the selection of the PTM for high-pressure experiments with a CN should take into account not only its hydrostatic limit and solubility but also the shape and the size of the molecules (dividing PTMs into penetrating and nonpenetrating subclasses).^34,46^ Indeed, for small-molecule PTMs, a counterintuitive effect of pressure-induced volume increase has been observed, compression driving PTMs to penetrate the pores and expanding the structure. Such effects were observed for ″breathing″ or flexible CNs such as the MOF ZIF-8.^43,47^ Other reports revealed that for CNs with sufficient flexibility, PTM inclusion can happen even if PTMs are larger than the pore windows of CNs.^48^

Alongside pressure-induced modification of the pore size and content, compression of CNs above 0.1 GPa can induce phase transitions,^49−51^ amorphization,^52,53^ or porosity generation by eliminating interpenetration.^54^ Furthermore, the structure of some CNs makes their compression anisotropic, and negative linear compressibility^55^ can be observed.^56−58^ In extreme cases, significant changes in crystal shape without destruction can occur.^59^ Other reports describe CNs that exhibit piezochromism, i.e., a color change induced by compression,^50,60^ which makes them applicable for pressure sensing. An unusual high-pressure behavior was observed for the ″edible″ γ-CD-MOF, an MOF based on molecules of γ-cyclodextrin, for which compression resulted in dissolution, an effect opposite to that expected. Moreover, by oscillating the pressure, the MOF was readily destroyed/resynthesized.^61^

Among the MOF subsets of the CSD,^39^ which comprises 112,400 entries, there are 761 deposits for structures investigated under pressure of at least 0.1 GPa. 3D MOFs, which might be expected to be more constrained by their structure compared to 1D and 2D MOFs, are the class of CNs most often studied by means of high-pressure crystallography, representing 62.42% of the CSD high-pressure MOF entries. Although 2D MOFs represent 24.78% of the MOF subset, they constitute only 13.80% of the high-pressure MOF subset, with 105 deposits for 15 distinct 2D CNs (i.e., having the same framework structure but different number and/or types of guests). Four CNs with **sql** topology^60,62−64^ are archived, which collectively represent 37 entries, and to the best of our knowledge, these CNs were not investigated for gas adsorption. Hence, it has not yet been confirmed whether they exhibit switching or not. Meanwhile, out of the 60 switching CNs reported from 2001 to 2020,^21^ only 3 were investigated under pressure of at least 0.1 GPa: MOF-508,^65^ MIL-53(Fe),^66^ and DUT-8(Ni).^67,68^ However, two of these materials, MOF-508 and DUT-8(Ni), were investigated with spectroscopic techniques supported by DFT calculations only. A Raman study of desolvated MOF-508b, with preheated NaCl used as the PTM, led to transformation into a form isomorphic with the open phase, MOF-508a, which underwent a subsequent transformation associated with the compression of the lattice at a pressure of approx. 1.5–2 GPa. Investigation of DUT-8(Ni) using Raman spectroscopy with MeOH as the PTM revealed that methanol molecules enable transition between closed and open phases at 0.16 GPa.^67^ Meanwhile, when DMF-loaded DUT-8(Ni) was studied with isopropanol used as the hydrostatic medium, solvent exchange was observed, but close-to-open transformation was not induced.^68^ The use of a nonpenetrating medium, a silicon oil, revealed that the transformation to a closed phase occurred at 0.5 GPa.^68^ MIL-53(Fe) loaded with water was the only CN investigated under high pressure using PXRD, at the presence of nonpenetrating silicon oil, resulting in compression of the unit-cell volume.

Herein, we present the first, to the best of our knowledge, high-pressure single-crystal X-ray diffraction study on the CN exhibiting switching behavior, providing information on the structural response of the framework to pressure exceeding 0.1 GPa. In this study, we investigate the effect of high pressure on the OX-loaded **sql** topology CN, sql-1-Co-NCS, which we have studied previously,^22^ to answer the following questions about its high-pressure behavior: (i) What effects are induced in the structure of sql-1-Co-NCS·4OX under high pressure? (ii) Can the high interlayer separation observed for sql-1-Co-NCS·4OX be further increased by means of pressure? (iii) Will crystals of sql-1-Co-NCS·4OX undergo pressure-induced phase transformation similar or different to that observed upon lowering temperature?

## 2 Experimental Section

### 2.1 Synthesis of Sql-1-Co-NCS·4OX

{[Co(4,4′-bipyridine)_2_(NCS)_2_]·4OX}_*n*_ (sql-1-Co-NCS·4OX) was prepared according to the previously reported method based on solvent diffusion.^22^ A 1:1 vol ratio mixture of EtOH and OX was carefully layered over 4,4′-bipyridine dissolved in 5 mL of OX, and a filtrated solution of Co(NCS)_2_ in 5 mL of ethanol was layered over the EtOH/OX layer. The solution was then left to stand for a few days before it yielded orange crystals of sql-1-Co-NCS·4OX. The crystals were collected by filtration and washed with OX.

### 2.2 Single Crystal X-ray Diffraction (SCXRD) Experiments

#### 2.2.1 Ambient Condition SCXRD

A single crystal of as-synthesized sql-1-Co-NCS·4OX was selected for X-ray diffraction experiments, which were performed with a Bruker D8 Quest diffractometer equipped with an IμS microfocus Cu K_α_ anode (λ = 1.54178 Å) and Apex-II detector. Data were collected, indexed, integrated, and scaled using APEX3;^69^ absorption correction was performed by a multiscan method using SADABS;^70^ and the space group was determined using XPREP^71^ implemented in APEX3.

#### 2.2.2 High-Pressure SCXRD

A single crystal of as-synthesized sql-1-Co-NCS·4OX was loaded into a 0.4 mm wide opening in a 0.3 mm thick steel gasket mounted in a modified Merrill–Bassett diamond anvil cell (DAC)^72^ alongside a small ruby chip used for pressure measurement,^73^ and a cellulose fiber was used to prevent crystal movement during the experiment. The so-prepared cell was filled with OX or 4:1 vol MeOH/EtOH. The crystal was gradually compressed until a pressure of 1.04 GPa was reached. The pressure inside the DAC was measured using a Photon Control Inc. spectrometer, affording an accuracy of 0.02 GPa, using the ruby fluorescence method.^73^ Measurements between 0.09(2) and 0.54(2) GPa were performed in the presence of OX as PTM (Figure S1); however, experiments were hindered by the crystallization of OX above 0.55 GPa. Therefore, two additional measurements, at 0.28(2) and 1.04(2) GPa, were performed with 4:1 vol MeOH/EtOH as a hydrostatic medium (Figure S2). All high-pressure X-ray diffraction experiments were performed with a four-circle Xcalibur diffractometer equipped with an EOS CCD detector and Mo K_α_ (λ = 0.71073 Å) X-ray tube. The CrysAlisPro^74^ program was used for data collection, determination of the *UB*-matrix, absorption correction, and data reduction.

### 2.3 Crystal Structure Solution and Refinement

All structures were solved with intrinsic phasing using ShelXT^75^ and refined with the least squares method using ShelXL^76^ and Olex2^77^ as an interface. Due to the high disorder of the guest molecules and the low quality of the collected data, which is limited by the construction of the DAC, the electron density associated with guest molecules present in the pores of the **sql** CN was omitted from refinement using a masking algorithm^78,79^ implemented in Olex2 (for solvent radius and truncation equal 1.9 and 1.2 Å, respectively). Nonhydrogen atoms were refined anisotropically, whereas hydrogen atoms were located from the molecular geometry at idealized positions with isotropic thermal parameters depending on the equivalent displacement parameters of their carriers. Crystallographic information for all reported crystal structures is listed in Table S1 in the Supporting Information. Structures were deposited in the Cambridge Crystallographic Data Centre (CCDC 2145115–2145122) and can be accessed free of charge by filling an online application form at https://www.ccdc.cam.ac.uk/structures/.

### 2.4 CSD Data Mining

The Cambridge Structural Database (v 5.43, November 2021), including MOF^39^ and ″High-pressure″ subsets, was data-mined using Python API^36^ to determine the dimensionality of MOFs and values of the dihedral angle between pyridine rings in 4,4′-bipiridine moieties. Additionally, a manual survey of the database using ConQuest^80^ was performed to analyze deposited structures of sql-1-M-NCS (M-metal) coordination networks.

The dimensionality of the MOFs was determined by analyzing the dimensions of the minimal ellipsoids that bound the expanded polymer frameworks.^81^ Therefore, only deposits with a complete set of coordinates and defined polymeric bonds were used in the analysis. Additionally, the high-pressure MOF subset was manually examined for validation, and it was shown that the proper dimensionality was assigned for 91.72% of the refcode families. Due to the high accuracy of the automatic analysis of the high-pressure MOF subset, possible discrepancies in the dimensionality assigned for deposits from the complete MOF subset were considered statistically negligible, as no systematic errors were detected. Therefore, all values discussed in this work that refer to the MOF subset of the CSD are obtained by automatic analysis. To assess the number of MOFs of **sql** topology investigated under pressure ≥0.1 GPa, 2D structures from the high-pressure MOF subset were manually analyzed using Mercury.^82^

### 2.5 Energy Calculations

The total electronic energy of 4,4′-bipyridine molecules of various conformations was obtained using the PSI4^83^ program as a Python module at the B3LYP/STO-3G level of theory. Calculations were made for 91 conformers generated by rotation of one of the pyridine rings about the C_4_-C_4′_ bond (with 1° step). For the purpose of our discussion, energy values are expressed relative to the energy minimum.

### 2.6 Crystal Structure Analysis

For analysis of the structural voids, the Mercury program^82^ was used. In all cases, the structural voids were calculated for the contact surface, with probe radius and grid spacing of 1.9 and 0.7 Å, respectively. The default value of the grid spacing was used, while the radius is half of the minimal dimension of the OX molecule after Webster *et al*.^84^ For calculating the void volume between **sql** networks (*V*_void-int_), the space within the grid was artificially filled by inserting *dummy* atoms (see Figure S3 and Table S2 in the Supporting Information). To establish the void volume within the grid (*V*_void-grid_), *V*_void-int_ was subtracted from the total void volume (*V*_void_; see Table S3).

## 3 Results and Discussion

### 3.1 Sql-1-Co-NCS·4OX at Ambient Conditions

At ambient conditions, crystals of OX-loaded sql-1-Co-NCS were found to exhibit tetragonal symmetry (space group *I*4/*mmm*), meaning that the adsorption of OX molecules at RT increased the symmetry of the crystal compared to the nonporous phase, which crystallized in the monoclinic space group *C*2/*c*.^31,32^ A previous report revealed that, upon lowering the temperature, the OX-loaded phase underwent a phase transformation to lower symmetry (monoclinic, space group *C*2/*c* at 100 K).^22^ Herein, we denote tetragonal sql-1-Co-NCS·4OX as Phase I and the monoclinic form as Phase II. Lowering the temperature to 100 K did not affect the symmetry of the closed phase of sql-1-Co-NCS.^15^

**Figure 1:**
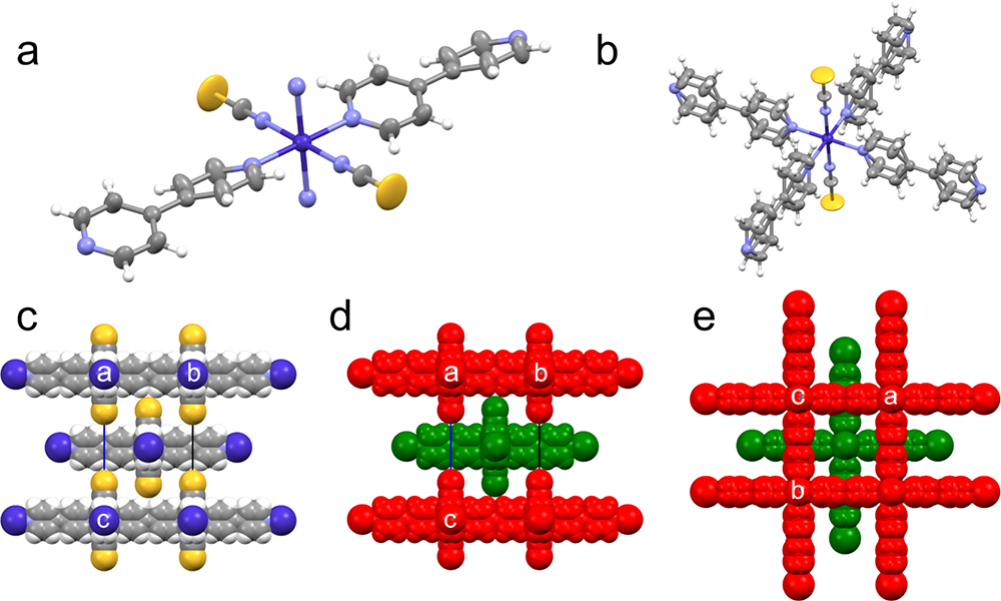
Coordination of the metal center in sql-1-Co-NCS·4OX at ambient conditions (a, b), shown (b) with and (a) without 4,4′-bipyridine ligand disorder, as well as the arrangement of the **sql** layers (c–e) along [100] (c, d) and [001] (e). In parts (d) and (e) of the figure, the layers were colored red and green to give a clearer representation of their arrangement.

Phase I crystals of sql-1-Co-NCS·4OX are composed of **sql** networks propagating in the (001) plane and stacked in the [001] direction. The coordination networks are composed of cobalt cations octahedrally coordinated by four 4,4′-bipyridine ligands in the equatorial positions and two thiocyanate anions in the axial positions (Figure 1a). The Co cation, thiocyanate anion, and 4,4′-bipyridine ligands lie on special positions of 4/*mmm*, 4*mm*, *mmm* symmetry, respectively. Additionally, due to their position, the 4,4′-bipyridine ligands are disordered by symmetry in a 1:1 ratio (Figure 1b). As a result, the asymmetric unit contains 1/16 of Co^2+^, 1/8 of NCS^–^, and 1/8 of the 4,4′-bipyridine ligand. It is not feasible to unequivocally assign the 4,4′-bipyridine ligand conformation, with the possibility of it having a random mixture of the planar and nonplanar conformations. The Co–Co–Co angle within the network, 90°, is the same as the Co–Co–Co angle between translation-related **sql** networks (Figure S4a). The thiocyanate anions point perpendicularly to adjacent nets directly in the center of the grid cavity (Figure 1c–e). Although determination of the position of the OX molecules within the framework was not possible due to high disorder, it can be assumed, based on the previous reports on adsorption and crystal structure of the sql-1-Co-NCS·4OX at 100 K,^22^ that OX molecules lie between the stacked **sql** networks and in the grid cavities.

The main difference between sql-1-Co-NCS·4OX Phase I and Phase II concerning the **sql** network is the Co–N–CS angle (180 vs 166° at 303 and 100 K, respectively; see Table 1). Otherwise, the networks do not differ significantly, with 4,4′-bipyridine ligands adopting a similar conformation. For the ligands of nonplanar conformation, the torsion angle is approx. 29–30° in both phases (Table 1). The presence of 4,4′-bipyridine ligands of planar conformation was confirmed in the structure at 100 K, and in Phase I, it is plausible, but confirmation is precluded by disorder and symmetry. The geometry of the grid is also similar, with square grid angles being equal to 90° in Phase I at RT (as expected due to symmetry), while in the monoclinic phase, it only slightly deviates from 90° (by *ca*. 0.02°). When the overall crystal structure is considered, transformation from the tetragonal to the monoclinic phase results in changes in the arrangement of the **sql** networks. The interlayer separation, expressed as the distance between two planes (calculated for four Co cations at the corners of the **sql** window of two neighboring **sql** layers, Figure S5) is approx. 0.3 Å (i.e., by 3.2%) greater for sql-1-Co-NCS·4OX Phase I at 303 K (Table 1). We attribute this to the increased thermal motion of the atoms of the framework and adsorbed OX molecules. Interestingly, for the closed sql-1-Co-NCS phase, increasing the temperature from 100 to 293 K increases the interlayer separation by only 0.02 Å (0.45%; Table 1). The relative positioning of the **sql** networks in Phases I and II of sql-1-Co-NCS·4OX is also different, with translation-related networks stacked in the [001] direction positioned orthogonally in Phase I consistent with tetragonal symmetry (Figure S4a) and at the angle of 63.75° in Phase II (for the **sql** networks stacked in the [100] direction, Figure S4b). The symmetry-related **sql** networks (i.e. networks at 1/2 and 1 *c_0_* in Phase I and at 1/2 and 1 *a_0_* in Phase II) also shift as a result of the phase transformation. The displacement can be expressed by an angle between the Co anion, the centroid calculated for two Co cations linked by 4,4′-bipyridine ligands, and the Co ion of the symmetry-related network (Table 1, Figure S6). In Phase I, the angle is 90° (at 303 K/0.1 MPa) in all directions, while in Phase II at 100 K/0.1 MPa, it ranges from 83.88 to 96.10° (Table 1). This displacement has an effect on the accessible space between the **sql** layers. Although the interlayer separation is higher in Phase I, the void space between the **sql** networks is smaller compared to Phase II at 100 K (Figure S7, Table S3). It appears that, upon lowering the temperature, thermal contraction of the structure decreases the interlayer separation, but this effect is compensated by the shift in the position of the layers. Furthermore, the angles between the pyridine rings of the 4,4′-bipyridine ligands and the plane of the **sql** network change on transformation from Phase I to Phase II to become more accommodating to the adsorbed OX molecules. In Phase II, the 4,4′-bipyridine ligands, on average, are more aligned with the plane of the **sql** layer (Table S4), as expressed by the angle between the plane calculated for four Co cations at the corners of the **sql** network window and the plane calculated for the pyridine ring of the 4,4′-bipyridine ligand (Figure S8). The pyridine rings are at angles of approx. 57 and 86° in the nonplanar 4,4′-bipyridine ligands and 67° in the planar 4,4′-bipyridine. Such an orientation allows for the formation of the C–H···π interactions between the framework and the OX molecules located between the **sql** layers and oriented parallel with respect to the framework plane (Figure S9a). In Phase I, the dihedral angle between the pyridine rings and the plane of the **sql** layer is *ca*. 75°, meaning that they protrude more in the [001] direction, limiting the interlayer space available to OX molecules.

**Table 1:** Structural Parameters for the Closed and OX-Loaded Phases of sql-1-co-NCS at Various Temperature and Pressure Conditions^a^

compound/refcode	SG	IS [Å]	square grid angles [°]	Co–N–CS angle [°]	4,4′-bipyridine dihedral angle [°]	Co–Co–Co angle [°]^b^	Co–Ctr–Co angle [°]^c^
sql-1-Co-NCS/YUVROX^32^(293 K/0.1 MPa)	*C*2/*c*	4.48	75.63/104.37	166.81	0/56.48 (1:1 ratio)	73.28/90.00	111.43/90.00/87.42/70.99
sql-1-Co-NCS/YUVROX01^15^(100 K/0.1 MPa)	*C*2/*c*	4.46	75.33/104.67	172.45	0/55.96 (1:1 ratio)	72.73/90.00	109.30/92.84/90.00/68.00
sql-1-Co-NCS·4OX II/KODDUF^22^(100 K/0.1 MPa)	*C*2/*c*	9.26	89.98/90.02	166.09	0/29.29 (1:1 ratio)	63.75/90.00	90.02/96.10/90.00/83.88
sql-1-Co-NCS·4OX I/*this study*(303 K/0.1 MPa)	*I*4*/mmm*	9.58	90	180	30.12^d^	90.00	90.00
sql-1-Co-NCS·*x*OX I*b*/*this study*(298 K/1.04 GPa)	*I*4*/mmm*	8.90	90	159.91	43.43^d^	90.00	90.00

a SG: space group; IS: interlayer separation.

b The angle between **sql** networks measured according to Figure S4.

c The angle between **sql** networks measured according to Figure S6; Ctr stands for a centroid calculated for two cobalt cations linked by the 4,4′-bipyridine ligand.

d The 4,4′-bipyridine lies in a special position (of *mmm* symmetry), which means that the 4,4′-bipyridine ligands can adapt the planar conformation as well.

### 3.2 Sql-1-Co-NCS·4OX under High Pressure

**Figure 2:**
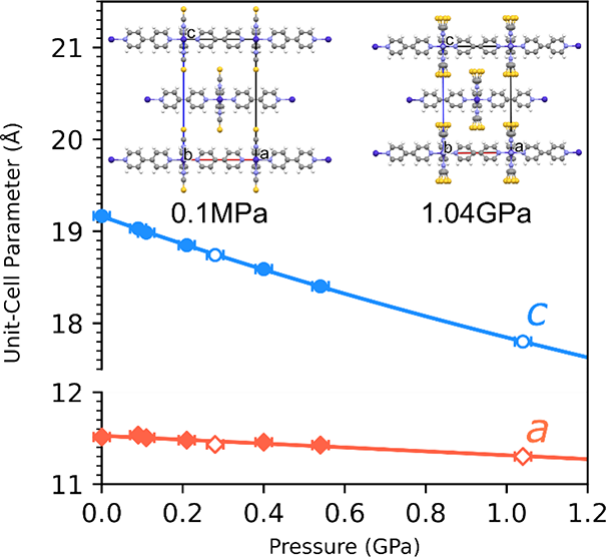
Pressure dependence of unit-cell parameters *a* (red diamonds) and *c* (blue circles) in compressed sql-1-Co-NCS·*x*OX crystals. Figures representing unit-cell packing, shown along [010], at two pressure extremes are included for comparison. The empty markers show data for experimental points where MeOH/EtOH was used as the hydrostatic medium. The error bars for the values of the unit-cell parameters are smaller than the size of the markers.

The tetragonal symmetry of space group *I*4*/mmm* of sql-1-Co-NCS·4OX Phase I crystals is preserved upon compression at least up to 1 GPa. The number of OX molecules for high-pressure structures is not indicated in the formula because it was not possible to locate guest OX molecules because of the disorder and low completeness of the high-pressure data, as well as the possibility that the number of adsorbed molecules per framework might vary with pressure. For both types of hydrostatic medium used, the compression of crystals of sql-1-Co-NCS·*x*OX Phase I leads to a gradual decrease of the unit-cell volume. The most significant change in the unit-cell dimensions was observed for the *c* axis (Figure 1), which decreased by almost 1.3 Å (6.8%), while for the *a* axis, only a slightly change, by about 0.1 Å, was observed (Figure 2). This is expected as *a* is limited by the size of the window in the **sql** network, which is constrained by covalent and coordination bonds. On the other hand, **sql** networks are stacked along [001], with structural voids filled by OX molecules. It appears that high pressure forces molecules out of the framework instead of forcing them into and further expanding the interlayer separation, as evident from the distance between the **sql** layers decreasing significantly, by 7%, from 0.1 MPa to 1 GPa (Table 1 and Table S4). Therefore, it seems that the expansion of sql-1-Co-NCS achieved under OX vapor (at relative pressure *P*/*P*_0_ ≈ 25%)^22^ had already reached the framework limit.

**Figure 3:**
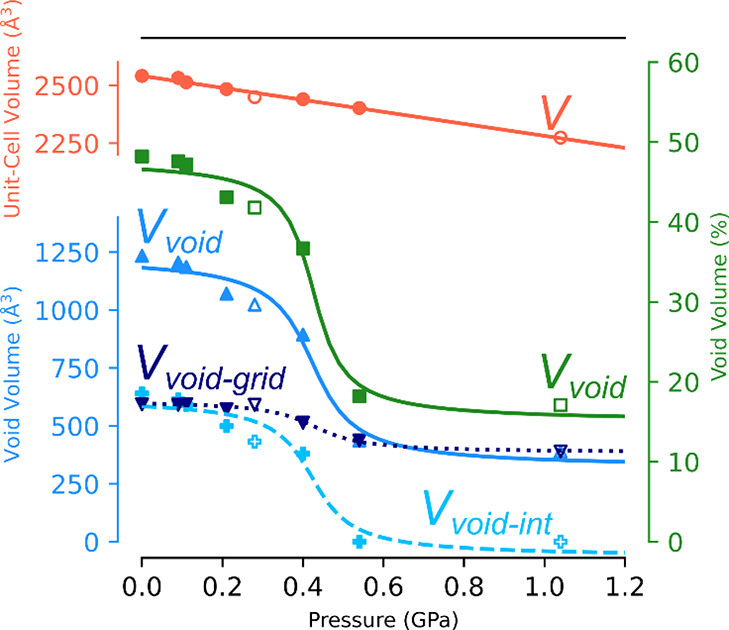
Pressure dependence of the unit-cell volume *V* (red circles) and void volume (expressed in Å^3^ and %, shown as blue triangles/pluses and green squares, respectively) in compressed sql-1-Co-NCS·xOX crystals. The appropriate scales are drawn in the corresponding colors. The total (*V*_void_), interlayer (*V*_void-int_), and grid cavity (*V*_void-grid_) void volumes, per unit cell, expressed in Å^3^ are marked with up-pointing triangle, plus and down-pointing triangle markers, as well as solid, dashed, and dotted lines, respectively (all in shades of blue). The lines representing functions fitted to the experimental points are for guiding the eye only. As the interlayer void volume decreases to 0 above 0.5 GPa, the axes of the graph were separated at the corners for clarity of the figure. The experimental points for MeOH/EtOH hydrostatic medium are shown with open markers.

Because OX molecules could not be located crystallographically, their release from the crystal was not straightforward to measure, but it is evident from the change in the volume of OX-accessible voids. The observed decrease in unit-cell volume was almost linear as it dropped by about 266 Å^3^ (10.5%) between 0.1 MPa and 1.04 GPa. However, the relative volume change in the voids occupied by OX is more significant (Figure 3 and Figure S10); initially, these voids represented almost 50% of the unit-cell volume, but on compression up to 1 GPa, they dropped to 15%. Interestingly, this compression was step-like. Initially, up to 0.3 GPa, it gradually decreased. Subsequently, between 0.3 and 0.5 GPa, a sudden drop in a void space available to OX was observed, from 1023 to 445 Å^3^. Above 0.5 GPa, the volume of the voids remained almost constant (Figure 3, Table S3). The interlayer voids *V*_void-int_ of a size sufficient to be occupied by OX molecules were lost during compression in the 0.3–0.5 GPa pressure range, the remaining voids being the grid cavities of the **sql** network (Table S2, Figure S3). For structures in which the space in the opening of the network was calculated, it can be shown that the remaining void space, *V*_void-int_, calculated for the contact surface (probe radius of 1.9 Å and grid spacing of 0.7 Å) dropped to 0 above 0.4 GPa (Figure 3, Figure S11, Table S3). Therefore, it can be presumed that, at this point, the adsorbed OX molecules located between the **sql** networks had been released. Concurrently, the difference between the voids calculated for the whole structure and for the interlayer space exclusively, i.e., voids associated with the cavities in the **sql** networks (*V*_void-grid_ = *V*_void_ – *V*_void-int_), shows that pressure had little effect upon the void space within the grid. The transformation of the crystal structure caused by the release of OX molecules from the framework is also evidenced macroscopically, as fractures on the faces of the crystal at 0.40 and 0.54 GPa had become noticeable (Figures S1 and S2).

**Figure 4:**
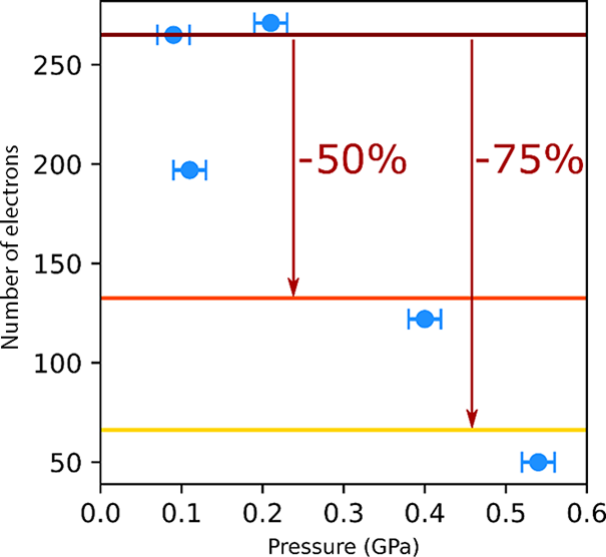
The electron count per unit cell masked during the refinement of sql-1-Co-NCS·*x*OX structures for OX experimental series as a function of pressure (blue circles). Horizontal red, orange, and yellow lines were added to mark the levels corresponding to 100, 50, and 25% of the electron count at 0.09 GPa.

Additionally, the desorption of OX molecules can be evidenced by the change in the number of electrons found in the pore volume masked during the structure refinement (Table 2). As shown in Table 2 and Figure 4, the electron count per unit cell decreases with pressure. However, it should be noted that the number of electrons should not be used to calculate the exact number of OX molecules per formula unit, as low completeness of data affects the calculation of the electron density located in the voids, making the number of masked electrons unreliable.^85^ However, use of the same sample crystal and similar data completeness (approx. 40%) for the experimental series measured in the presence of OX as the hydrostatic medium allow one to observe the relative change in the pore content and to correlate it with changes observed in the void volume. Data collected for the experiment with 4:1 MeOH/EtOH as PTM were excluded from the analysis, as it is plausible that methanol and ethanol molecules replaced OX molecules, affecting the composition of the pores and therefore the electron count. It can be observed that for the structure at 0.4 GPa, the number of electrons dropped by approx. 50% relative to the initial value at 0.09 GPa. If it is assumed that at the lowest pressure for the OX-series the framework is fully loaded with OX molecules, i.e., four molecules of OX present per formula unit, at 0.4 GPa, the amount of the OX in pores drops to two per formula unit. When this is contrasted with the change in the volume of the interlayer voids (*V*_void-int_) that drops to 380.21 Å^3^, i.e., by approx. 40% in respect to the structure at 0.09 GPa (Figure 3, Table S3), it can be assumed that the desorbed molecules come from the pores between the **sql** layers. Increasing pressure to 0.54 GPa led to a further decrease in the number of electrons found in the masked void volume, now dropping below 25% of the value at 0.9 GPa, also accompanied by the total contraction of interlayer voids and changes in the conformation of the **sql** network. These data indicate that the desorption from the **sql** network cavities had occurred.

**Table 2:** Void Volume and Number of Electrons per Unit Cell Masked by the Masking Algorithm of the Olex2 Program

pressure (GPa)	masked void volume (Å)	masked void electron count
0.09(2)	957.0	265
0.11(2)	940.5	197
0.21(2)	887.5	271
0.40(2)	797.3	122
0.54(2)	131.4	25
	131.4	25

**Figure 5:**
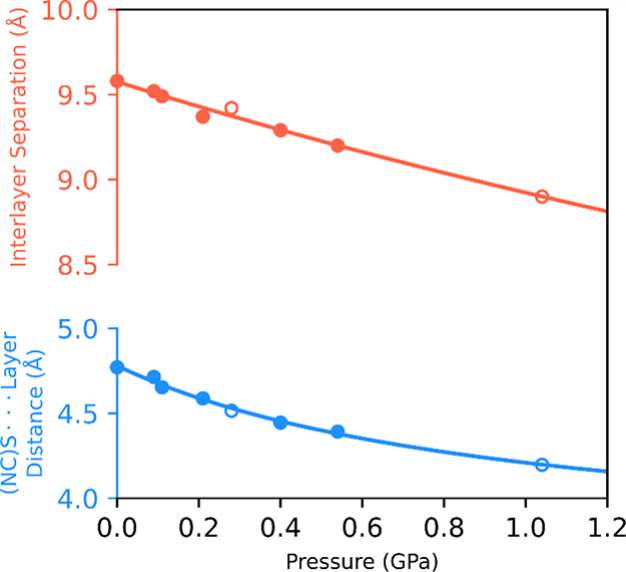
Pressure-induced changes in the interlayer separation (red) and the distance between the sulfur atom of the thiocyanate anion and the **sql** network plane (blue). The empty markers show data for experimental points where MeOH/EtOH was used as a hydrostatic medium.

Interestingly, despite the decrease in the interlayer separation higher than that induced by temperature (7 vs 3%), no phase transformation associated with the symmetry change was observed. This is probably because lowering the temperature did not induce the release of OX molecules from the framework, meaning that the structure adapted by undergoing a phase transition. Meanwhile, compression of sql-1-Co-NCS·*x*OX affected the content of the pores to release the strain that arises under high pressure. Still, although the space between the layers became too small for OX molecules, the interlayer separation did not decrease to the level observed for the closed phase of sql-1-Co-NCS (Figure 5, Table 1), remaining higher than for most reported sql-1-M-NCS·guest structures (Table S5). Only for sql-1-Co-NCS CNs loaded with xylenes was the interlayer spacing higher, exceeding 9 Å. This may indicate that OX molecules (or a mixture of OX, EtOH, and MeOH) adsorbed within the grid are still present and were not fully evacuated following compression at 1.04 GPa. Indeed, for the structure at 0.54 GPa, the electron count is associated with **sql** cavities exclusively, as only the voids in these cavities were masked (Figure S12). Since the number of electrons has dropped by more than 75% with respect to the structure at 0.09 GPa, it appears that the **sql** cavities were partially emptied; however, guest molecules must have remained in some sections of the coordination network, prohibiting the closure of the structure. For the occupied **sql** cavities, if the orientation of the OX molecules within the grid is assumed to be similar to that observed for sql-1-Co-NCS·4OX, the presence of adsorbed molecules would hinder compressions of the **sql** layers as thiocyanate anions would not be able to penetrate the opening of the neighboring grid. Indeed, this was observed in the pressure-induced changes through the distance between the sulfur atom of the thiocyanate anion (oriented perpendicularly toward the opening in the **sql** networks in Phase I at ambient conditions) and the plane of the **sql** layer (calculated for the Co cations of the **sql** network, see Figure S13). This distance decreases with pressure (Figure 5) and at 0.4 GPa is less than the distance observed for sql-1-Co-NCS·4OX Phase II at 100 K (4.445 vs 4.492 Å). Above 0.4 GPa, NCS anions become disordered by symmetry (Figure 2) and no longer point perpendicularly toward the grid, with the Co–N–CS angle deviating from 180°, as seen in the structures at 0.54 and 1.04 GPa (Table S4). This change in the orientation of the thiocyanate anion enabled further contraction of the **sql** networks because it precludes the need to infiltrate the OX-occupied cavities in the grid. It is possible that the reduction in the Co–N–CS angle from 180° starts at a pressure lower than 0.54 GPa as the thermal ellipsoids of the S and C atoms of the thiocyanate anions become wider; however, due to the location of the NCS anion in a special position leading to disorder by symmetry, and presumably only a slight deviation from 180°, modeling of the disorder was not possible for the high-pressure data between 0.09 and 0.4 GPa. Therefore, the structure was refined with all atoms of the NCS anion lying on the special position with 4*mm* symmetry. Only when the departure of the Co–N–CS angle from 180° became significant (i.e., for structures at 0.54 and 1.04 GPa) could the disorder be modeled.

Based on the changes in the electron count, void volume, and orientation of thiocyanate anions, two partially loaded intermediate phases are proposed. One, which exists in 0.3–0.5 GPa range, can be considered to be half-loaded OX Phase I, i.e., Phase I*a*. Phase I*b* was observed at 0.54 GPa, almost empty of guest molecules, but, due to the high interlayer separation, cannot be considered as a closed phase. Although crystals measured in the presence of MeOH/EtOH were excluded from the analysis of the pore composition, based on the geometry of the framework and crystal packing, the structure at 0.28 GPa can be assigned to Phase I*a* and that at 1.04 GPa to Phase I*b*. It is worth noting that for sql-1-M-NCS coordination networks, one-step sorption isotherms are usually observed, with some exceptions: CO_2_-loaded sql-1-Fe-NCS (at 195 K) and EB-, MX-, and PX-loaded sql-1-Co-NCS (at 298 K) exhibited two-step desorption.^22,30^ Indeed, out of the all xylene loaded sql-1-Co-NCS crystals, only for the OX-loaded phase was a one-step desorption noted.^22^ That the pressure-induced desorption of sql-1-Co-NCS·*x*OX proceeded via at least two partially loaded intermediate phases resembles ELM-11, which exhibited multistep CO_2_ and C_2_H_2_ sorption isotherms at 195 K.^28^

**Figure 6:**
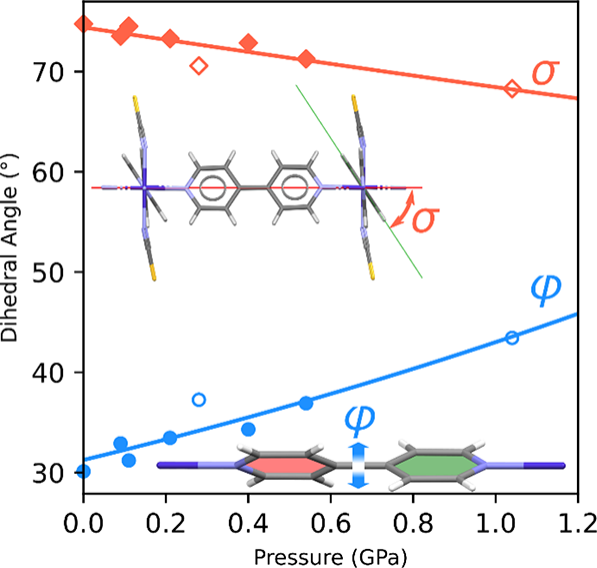
Pressure-induced changes in the 4,4′-bipyridine dihedral angle φ of nonplanar conformation (blue circles) and dihedral angle between the pyrimidine ring of 4,4′-bipyridine and the **sql** network plane σ (red diamonds). The empty markers show data for experimental points where MeOH/EtOH was used as a hydrostatic medium. Figures of fragments of the **sql** network were included to illustrate how angles φ and σ were measured.

**Figure 7:**
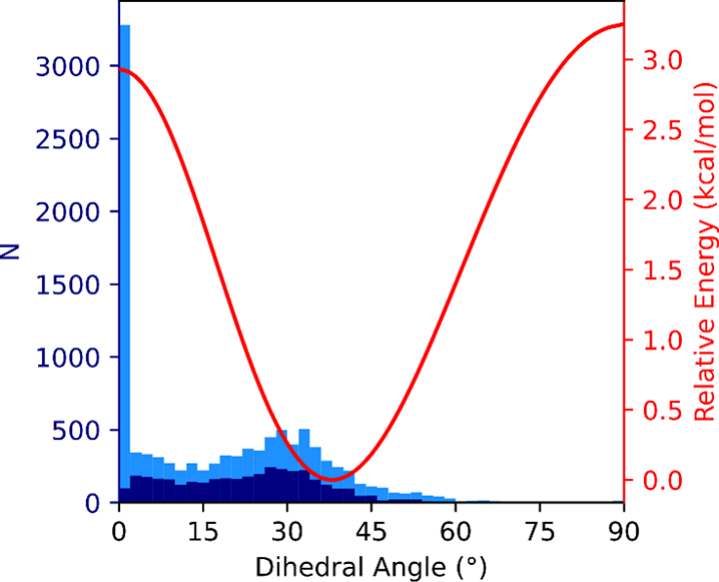
Stacked histogram showing the number of 4,4′-bipyridine entities of a specific dihedral angle between the planes of the pyridine rings present in the CSD deposits (bin width equals to 2°). The navy blue bars represent the 4,4′-bipyridine molecules located in general positions (i.e., a position when molecule is being mapped onto itself only by the identity operation^86^), while light blue bars represent those in special positions (i.e., when it is being mapped onto itself by the identity operation and by at least one other symmetry operation of the space group^86^). The red solid line marks the total electronic energy values for 4,4′-bipyridine conformers (in reference to the minimum) against the dihedral angle between planes of pyridine rings in the isolated molecule.

Increased pressure also affected the conformation of 4,4′-bipyridine ligands. The angle between the pyridine rings of the ligands and the **sql** plane decreased at 1.04 GPa, dropping below 70° (Table S4). Therefore, it appears that a similar effect to that observed for lowering temperature was achieved, where the 4,4′-bipyridine ligands became more aligned with the **sql** plane. Concurrently, the conformation of the nonplanar 4,4′-bipyridine ligands started to deviate from planarity. Initially, in Phase I at ambient conditions, the dihedral angle between the planes of pyridyl rings is *ca*. 30°, close to the population maximum found for the structures deposited in CSD (Figure 6). Meanwhile, at 1.04 GPa, it exceeds 40° (Figure 6, Table S4), which is close to the calculated energy minimum of 38° (Figure 7) but corresponds to a lower number of reported structures.

## 4 Conclusions

The OX-loaded sql-1-Co-NCS was investigated in the 0.1 MPa–1 GPa pressure range. The crystal structure of sql-1-Co-NCS·4OX at ambient conditions was found to be different from the known structure at 100 K. Therefore, the existence of two polymorphs, Phase I and II, of sql-1-Co-NCS·4OX has been established. The main difference between these forms is the relative positioning of the **sql** networks, which is reflected in the symmetry of the crystals. Although it was shown that lowering the temperature can lead to a reduced symmetry in sql-1-Co-NCS·4OX crystals, exposure to high pressure did not induce the same effect. Despite the ability of sql-1-Co-NCS to intercalate OX molecules, when OX-loaded sql-1-Co-NCS was exposed to pressure exceeding 0.1 GPa, no additional OX molecules were adsorbed. Instead, the space between the **sql** layers decreased, and changes in the visual appearance of the crystal were observed. Moreover, compression of sql-1-Co-NCS·4OX crystals affected the conformation of the 4,4′-bipyridine ligands, the position of the thiocyanate anions with respect to the grid, interlayer voids, and the electron density in the pores. We attribute these observations to the partial desorption of guest molecules, and on the basis of our structural analyses, it can be concluded that the high pressure induced multistep release of OX molecules via two intermediate partially loaded phases, I*a* and I*b*. Conversely, lowering the temperature to 100 K resulted in a decrease in interlayer separation with the content of the pores preserved. Rather, strain was addressed by changing the relative positioning of the layers during phase transition to monoclinic Phase II.

## Supporting Information Available

Tables listing (i) crystallographic and experimental information, (ii) structural information concerning the geometry of the studied compound, (iii) structural void parameters, and (iv) brief summary of CSD and literature survey of the reported sql-1-M-NCS structures; figures of sample crystals; and figures supporting the discussion (i.e., showing the method for angles, distances, and void calculation) (PDF)

## Abbreviations

CN: coordination network
OX: *o-*xylene
PX: *p-*xylene
MX: *m-*xylene
EB: ethylbenzene
