# Supplementary material for: Pressure-Induced Structural Effects in the Square Lattice (sql) Topology Coordination Network Sql-1-Co-NCS·4OX
Source: Cryst Growth Des. 2022 Oct 31;23(4):2055–64. doi: 10.1021/acs.cgd.2c00982 (PMC10080653; doi:10.1021/acs.cgd.2c00982)
Supplement: Supplementary file 1 — cg2c00982_si_001.pdf [file cg2c00982_si_001.pdf]

Supporting Information

Pressure induced structural effects in the square lattice (sql) topology coordination network sql-1-Co-NCS·4OX

Ewa Patyk-Kaźmierczak,[a]* Michał Kaźmierczak,[a] Shi-Qiang Wang,[b] Michael J. Zaworotko[b]

[a] Department of Materials Chemistry, Faculty of Chemistry, Adam Mickiewicz University in Poznań, Uniwersytetu Poznańskiego 8, 61-614, Poznań, Poland.
[b] Department of Chemical Sciences and Bernal Institute, University of Limerick, Co. Limerick, V94T9PX, Ireland.

*e-mail address: ewapatyk@amu.edu.pl

Table of content

1. Tables 2
  1.1 Crystallographic and refinement details 2
  1.2 Structural voids 4
  1.3 Framework geometry 5
2. Figures 5
  2.1. Sample crystals 5
  2.2. Crystal structure analysis 6
3. References 11

1. Tables

1.1 Crystallographic and refinement details

Table S1. Crystallographic and structure refinement information for OX-loaded sql-1-Co-NCS crystals.

name	sql_ox_RT	sql_ox_90MPa	sql_ox_110MPa	sql_ox_210MPa
Formula*	$C_{22}H_{16}CoN_6S_2$	$C_{22}H_{16}CoN_6S_2$	$C_{22}H_{16}CoN_6S_2$	$C_{22}H_{16}CoN_6S_2$
Phase	I	I	I	I
Pressure (GPa)	0.0001	0.09(2)	0.11(2)	0.21(2)
Temperature (K)	303(2)	298(2)	298(2)	298(2)
Formula weight*	487.46	487.46	487.46	487.46
Crystal colour	Orange	Orange	Orange	Orange
Crystal size (mm)	0.20x0.20x0.05	0.21x0.17x0.15	0.21x0.17x0.15	0.21x0.17x0.15
Hydrostatic medium	None	*o*-xylene	*o*-xylene	*o*-xylene
Crystal system	Tetragonal	Tetragonal	Tetragonal	Tetragonal
Space group	*I4/mmm*	*I4/mmm*	*I4/mmm*	*I4/mmm*
Unit cell dimensions (Å; º)				
a =	11.5125(3)	11.534(2)	11.5047(8)	11.4789(10)
c =	19.1668(6)	19.031(12)	18.986(6)	18.849(8)
Volume (Å^3)	2540.32(15)	2531.9(18)	2512.9(8)	2483.6(11)
Z	2	2	2	2
D_x (g cm^{-3})*	0.637	0.639	0.644	0.652
Wavelength MoKα, λ (Å)	1.54178	0.71073	0.71073	0.71073
Absorption coefficient (mm^{-1})	3.493	0.431	0.434	0.439
F(000) (e)*	498	498	498	498
2θ max (º)	119.98	49.25	49.52	49.65
Min./Max. indices				
h	-10/12	-10/10	-13/13	-11/11
k	-12/12	-13/13	-10/11	-13/13
l	-16/21	-14/14	-13/13	-13/13
Reflections collected/unique	5161/580	2864/240	3229/263	3149/256
R_{int}	0.0350	0.1040	0.1085	0.1121
Observed reflections (I>4σ(I))	573	170	182	178
Data/parameters	580/38	240/38	263/38	256/38
Goodness of fit	1.152	1.020	1.070	1.011
Final R_1/wR_2 indices (I>4σ(I))	0.0749/ 0.2222	0.0389/ 0.0930	0.0431/0.0932	0.0438/0.0907
R_1/wR_2 indices (all data)	0.0757/ 0.2247	0.0557/0.0971	0.0652/0.1011	0.0627/0.0961
$\Delta\rho_{max}$, $\Delta\rho_{min}$ (eÅ^{-3})	0.833/ -0.471	0.076, -0.153	0.111/-0.107	0.138/-0.103
Weighting scheme[a]: x; y	0.181200; 0.841200	0.0539; 0	0.0534;0	0.0512;0
Extinction coefficient	–	–	–	–
Absorption correction type	multi-scan	multi-scan	multi-scan	multi-scan
T_{min}/T_{max}	0.5457/0.7518	0.57 / 1.00	0.77/1.00	0.83/1.00

*Please note that these values are given for the framework alone, not taking into account the adsorbed *o*-xylene molecules as their exact number and position in the structure could not be determined.

Table S1. Crystallographic and structure refinement information for OX-loaded sql-1-Co-NCS crystals.- *Continuation*

name	sql_ox_280MPa	sql_ox_400MPa	sql_ox_540MPa	sql_ox_1040MPa
Formula*	$C_{22}H_{16}CoN_6S_2$	$C_{22}H_{16}CoN_6S_2$	$C_{22}H_{16}CoN_6S_2$	$C_{22}H_{16}CoN_6S_2$
Phase	I*a*	I*a*	I*b*	I*b*
Pressure (GPa)	0.28(2)	0.40(2)	0.54(2)	1.04(2)
Temperature (K)	298(2)	298(2)	298(2)	298(2)
Formula weight*	487.46	487.46	487.46	487.46
Crystal colour	Orange	Orange	Orange	Orange
Crystal size (mm)	0.21x0.17x0.15	0.21x0.18x0.15	0.21x0.18x0.15	0.21x0.18x0.15
Hydrostatic medium	MeOH:EtOH	*o*-xylene	*o*-xylene	MeOH:EtOH
Crystal system	Tetragonal	Tetragonal	Tetragonal	Tetragonal
Space group	*I4/mmm*	*I4/mmm*	*I4/mmm*	*I4/mmm*
Unit cell dimensions (Å; °)				
a =	11.4317(10)	11.4564(10)	11.4251(8)	11.3010(14)
c =	18.742(19)	18.589(8)	18.401(8)	17.80(3)
Volume ($Å^3$)	2449(3)	2439.8(12)	2401.9(11)	2274(4)
Z	2	2	2	2
D_x (g cm^{-3})*	0.661	0.664	0.674	0.712
Wavelength MoKα, λ (Å)	0.71073	0.71073	0.71073	0.71073
Absorption coefficient (mm^{-1})	0.445	0.447	0.454	0.480
F(000) (e)*	498	498	498	498
2θ max (°)	49.87	49.87	49.94	49.42
Min./Max. indices				
h	-13/13	-11/11	-13/13	-12/12
k	-12/12	-13/13	-11/11	-13/13
l	-9/9	-12/12	-12/12	-9/9
Reflections collected/unique	3252/275	3209/274	3129/270	3502/275
R_{int}	0.1514	0.1182	0.1264	0.2667
Observed reflections (I>4σ(I))	156	185	200	118
Data/parameters	275/38	274/38	270/47	275/49
Goodness of fit	0.951	1.014	1.091	1.152
Final R_1/wR_2 indices (I>4σ(I))	0.0459/0.0706	0.0602/0.1340	0.0860/0.2167	0.1254/0.3304
R_1/wR_2 indices (all data)	0.0948/0.0795	0.0922/0.1448	0.1171/0.2367	0.2560/0.4168
$\Delta\rho_{max}$, $\Delta\rho_{min}$ (e$Å^{-3}$)	0.095/-0.128	0.174, -0.138	0.476/-0.299	0.462/-0.333
Weighting scheme[a]: x; y	0.0248; 0	0.0849; 0	0.1524;1.3257	0.2000;0
Extinction coefficient	–	–	–	–
Absorption correction type	multi-scan	multi-scan	multi-scan	multi-scan
T_{min}/T_{max}	0.93/1.00	0.80 / 1.00	0.75/1.00	0.79/1.00

*Please note that these values are given for the framework alone, not taking into account the adsorbed *o*-xylene molecules as their exact number and position in the structure could not be determined.

1.2 Structural voids

Table S2. Coordinates of the dummy atoms inserted in structures of sql-1-Co-NCS·4OX Phases I, I*a*, I*b* and II.

Phases I, I*a*, I*b*			Phase II					
x	*y*	*x*	*x*	*y*	*z*	*x*	*y*	*z*
0	0.6	0	0	0.1	0	0	0.1	0.15
0	0.7	0	0	0.2	0	0	0.2	0.15
0	0.8	0	0	0.3	0	0	0.3	0.15
0	0.9	0	0	0.4	0	0	0.4	0.15
0	1.0	0	0	0.5	0	0	0.5	0.15
0.1	0.6	0	0	0.6	0	0	0.6	0.15
0.1	0.7	0	0	0.7	*0*	0	0.7	0.15
0.1	0.8	0	0	0.8	0	0	0.8	0.15
0.1	0.9	0	0	0.9	0	0	0.9	0.15
0.1	1.0	0	0	0.1	0.05	0	0.1	0.2
0.2	0.6	0	0	0.2	0.05	0	0.2	0.2
0.2	0.7	0	0	0.3	0.05	0	0.3	0.2
0.2	0.8	0	0	0.4	0.05	0	0.4	0.2
0.2	0.9	0	0	0.5	0.05	0	0.5	0.2
0.2	1.0	0	0	0.6	0.05	0	0.6	0.2
0.3	0.6	0	0	0.7	0.05	0	0.7	0.2
0.3	0.7	0	0	0.8	0.05	0	0.8	0.2
0.3	0.8	0	0	0.9	0.05	0	0.9	0.2
0.3	0.9	0	0	0.1	0.1			
0.3	1.0	0	0	0.2	0.1			
0.*4*	0.6	0	0	0.3	0.1			
0.4	0.7	0	0	0.4	0.1			
0.4	0.8	0	0	0.5	0.1			
0.4	0.9	0	0	0.6	0.1			
0.4	1.0	0	0	0.7	0.1			
			0	0.8	0.1			
			0	0.9	0.1			

Table S3. Structural voids volume for structures of sql-1-Co-NCS·*x*OX Phases I, I*a* and I*b* in 0.1 MPa-1.04 GPa pressure range and at RT, and Phase II at 100 K/0.1 MPa (calculated for contact surface, probe radius= 1.9 Å, grid spacing= 0.7 Å).

REFCODE	p	T	V_{void}		$V_{void\text{-}int}$		$V_{void\text{-}grid}$	
			[Å^3]	[%]	[Å^3]	[%]	[Å^3]	[%]
KODDUF[1]	0.1 MPa	100 K	2813.06	57.9	1647.40	33.9	1165.66	24.0
This study	0.1 MPa	303 K	1223.70	48.2	655.49	25.8	568.21	22.4
This study	0.09 GPa	298 K	1189.54	47.0	630.75	24.9	558.79	22.1
This study	0.11 GPa	298 K	1175.73	46.8	603.70	24.0	572.03	22.8
This study	0.21 GPa	298 K	1001.99	40.3	499.08	20.1	502.91	20.2
This study	0.28 GPa	298 K	1023.28	41.8	492.18	20.1	531.10	21.7
This study	0.40 GPa	298 K	886.74	36.3	380.21	15.6	506.53	20.7
This study	0.54 GPa	298 K	444.50	18.5	0	0	444.50	18.5
This study	1.04 GPa	298 K	418.72	18.4	0	0	418.72	18.4

1.3 Framework geometry

Table S4. Structural information for sql-1-Co-NCS·*x*OX Phases I, I*a* and I*b* in 0.1 MPa-1.04 GPa pressure range and at RT, and Phase II at 100 K/0.1 MPa.

REFCODE	p/T	Interlayer separation [Å]	Co–N–CS angle [°]	Bipy torsion angle [°]	Dihedral angle (**sql** network - bipy pyridine ring) [°]	NCS – layer distance [Å]
KODDUF[1]	0.1 MPa/100 K	9.26	166.09	29.29/0 (1:1 ratio)	56.77/86.06/67.42 (1:1:2 ratio)	4.492
This study	0.1 MPa/303 K	9.58	180	30.12	74.75	4.771
This study	0.09 GPa/298 K	9.52	180	32.91	73.53	4.714
This study	0.11 GPa/298 K	9.49	180	31.21	74.52	4.653
This study	0.21 GPa/298 K	9.37	180	33.46	73.28	4.588
This study	0.28 GPa/298 K	9.42	180	37.26	70.57	4.515
This study	0.40 GPa/298 K	9.29	180	34.31	72.85	4.445
This study	0.54 GPa/298 K	9.20	163.48	36.91	71.26	4.392
This study	1.04 GPa/298 K	8.90	159.91	43.43	68.28	4.197

1.4 Summary of the reported sql-1-M-NSC structures

Table S5. Summary of CSD and literature survey of the reported sql-1-M-NCS structures

(where M=metal).

REFCODE	Formula	*p* (MPa)	*T* (K)	Guest compound	Interlayer separation	Closed/ open	3D structure?	Result of gas adsorption?	Ref.
**sql-1-Co-NCS**									
YUVROX	$(C_{22}H_{16}CoN_6S_2)_n$	0.1	293	-	4.48	Closed	Yes	N.A.	[2]
YUVROX01	$(C_{22}H_{16}CoN_6S_2)_n$	0.1	100	-	4.46	Closed	Yes	N.A	[3]
KODDUF	$(C_{22}H_{16}CoN_6S_2)_n{\cdot}4n(C_8H_{10})$	0.1	100	*o*-xylene	9.26	Open	Yes	Yes	[1]
KODFAN	$(C_{22}H_{16}CoN_6S_2)_n{\cdot}4n(C_8H_{10})$	0.1	100	*m*-xylene	9.23	Open	Yes	Yes	[1]
KODFER	$(C_{22}H_{16}CoN_6S_2)_n{\cdot}4n(C_8H_{10})$	0.1	100	*p*-xylene	9.23	Open	Yes	Yes	[1]
KODFIV	$(C_{22}H_{16}CoN_6S_2)_n{\cdot}2n(C_8H_{10})$	0.1	100	Ethylbenzene	6.25	Open	Yes	Yes	[1]
RINPUZ	$(C_{22}H_{16}CoN_6S_2)_n{\cdot}2n(C_4H_{10}O)$	0.1	283-303	Diethyl ether	6.49	Open	Yes	No	[4]
VIBQOP	$(C_{22}H_{16}CoN_6S_2)_n{\cdot}2n(C_7H_5F_3)$	0.1	273	(Trifluoromethyl)benzene	6.63	Open	Yes	No	[3]
VIBQUV	$(C_{22}H_{16}CoN_6S_2)_n{\cdot}3n(CO_2)$	0.1	283-303	Carbon dioxide	5.43	Open	Yes	Yes	[3]
**sql-1-Fe-NCS**									
ETOFAW	$(C_{22}H_{16}FeN_6S_2)_n{\cdot}2n(S_8)$	0.1	293	Octasulfur	7.29	Open	Yes	No	[5]
MUZKIC	$(C_{22}H_{16}FeN_6S_2)_n{\cdot}2n(CH_4O)$	0.1	170	Methanol	4.52	Closed	Yes	No	[6]
QAGVUR	$(C_{22}H_{16}FeN_6S_2)_n{\cdot}2n(C_2HCl_3)$	0.1	100	1,1,2-trichloroethene	5.87	Open	Yes	No	[7]
QAGWAY	$(C_{22}H_{16}FeN_6S_2)_n{\cdot}2n(C_7H_8)$	0.1	100	Toluene	5.92	Open	Yes	No	[7]
QAGWEC	$(C_{22}H_{16}FeN_6S_2)_n{\cdot}2n(C_6H_5NO_2)$	0.1	100	Nitrobenzene	6.19	Open	Yes	No	[7]
QAGWIG	$(C_{22}H_{16}FeN_6S_2)_n{\cdot}2n(C_4H_{10}O)$	0.1	100	Diethyl ether	6.28	Open	Yes	No	[7]
QAGWOM	$(C_{22}H_{16}FeN_6S_2)_n{\cdot}4n(C_3H_6O)$	0.1	100	Acetone s	7.04	Open	Yes	No	[7]
QAGWUS	$(C_{22}H_{16}FeN_6S_2)_n{\cdot}3n(CH_4O)$	0.1	100	Methanol	5.42	Open	Yes	No	[7]
QAGXAZ	$(C_{22}H_{16}FeN_6S_2)_n{\cdot}2n(CH_3NO_2)$	0.1	200	Methanol nitromethane	5.42	Open	Yes	No	[7]
QAGXED	$(C_{22}H_{16}FeN_6S_2)_n{\cdot}3n(CS_2)$	0.1	200	Carbon disulfide	5.96	Open	Yes	No	[7]
QAQTOT	$(C_{22}H_{16}FeN_6S_2)_n{\cdot}2n(CHCl_3)$	0.1	120	Chloroform	7.65	Open	Yes	No	[8]
TAPLIJ	$(C_{22}H_{16}FeN_6S_2)_n$	0.1	195	-	4.47	Closed	Yes	N.A	[9]
TAPLOP	$(C_{22}H_{16}FeN_6S_2)_n{\cdot}3n(CO_2)$	0.1	195	Carbon dioxide	5.42	Open	Yes	Yes	[9]

N.A.- not applicable; I.D.- Insufficient data; CCDC- CCDC communication

Table S5. Summary of CSD and literature survey of the reported sql-1-M-NCS structures (where M=metal)- *continuation*.

REFCODE	Formula	*p* (MPa)	*T* (K)	Guest compound	Interlayer separation	Closed/ open	3D structure?	Result of gas adsorption?	Ref.
**sql-1-Ni-NCS**									
CIZFOG	$(C_{22}H_{16}NiN_6S_2)_n$	0.1	283-303	-	4.46	Closed	Yes	N.A	[10]
CIZFOG01	$(C_{22}H_{16}NiN_6S_2)_n$	0.1	283-303	-	4.46	Closed	Yes	N.A	[11]
TAPLUV	$(C_{22}H_{16}NiN_6S_2)_n{\cdot}3n(CO_2)$	0.1	195	Carbon dioxide	5.44	Open	Yes	Yes	[9]
-	$(C_{22}H_{16}NiN_6S_2)_n{\cdot}4n(C_2H_2)$	0.05	195	Acetylene	I.D.	Open	No	Yes	[12]
**sql-1-Cu-NCS**									
IDOWAC	$(C_{22}H_{16}CuN_6S_2)_n$	0.1	273	Unknown solvate	6.64	I.D.	I.D.	No	CCDC
**sql-1-Mn-NCS**									
QUXLUR	$(C_{22}H_{16}MnN_6S_2)_n{\cdot}2n(C_4H_{10}O)$	0.1	230	Diethyl ether	6.43	Opened	Yes	No	[13]

N.A.- not applicable; I.D.- Insufficient data; CCDC- CCDC communication

2. Figures

2.1. Sample crystals

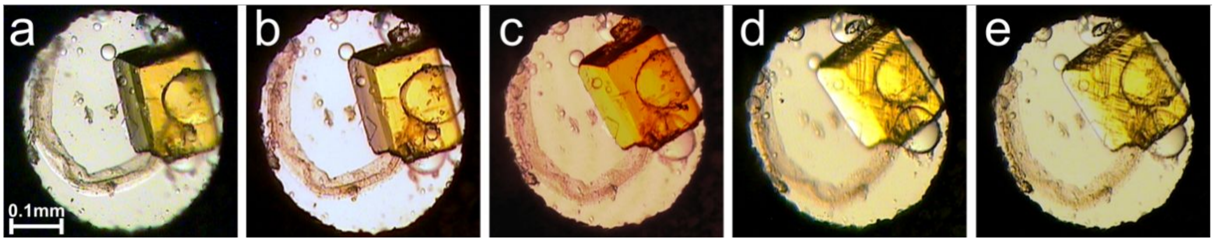

Figure S1. Single crystal of sql-1-Co-NCS·*x*OX in o-xylene (next to the right rim of the gasket opening) at (a) 0.09(2) GPa, (b) 0.11(2) GPa, (c) 0.21(2) GPa, (d) 0.40(2) GPa and (e) 0.54(2) GPa. A cellulose fiber used to fix position of the crystal is visible in the central section of the gasket opening, and ruby chip used for pressure measurement is placed above the sample crystal.

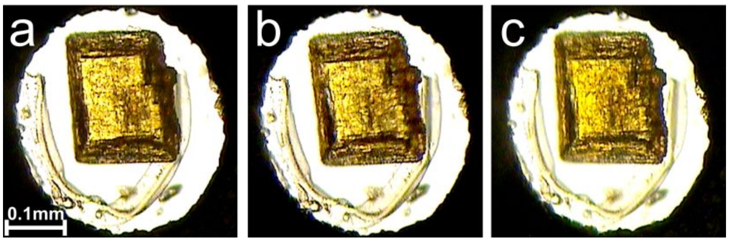

Figure S2. Single crystal of sql-1-Co-NCS·*x*OX in MeOH:EtOH (4:1 vol.), placed in the center of the gasket opening at (a) 0.14(2) GPa, (b) 0.21(2) GPa, (c) 1.04 (2) GPa. A cellulose fiber used to fix position of the crystal is visible in the central section of the gasket opening, and ruby chip used for pressure measurement is placed in the lower part of the gasket opening, next to the cellulose fiber.

2.2. Crystal structure analysis

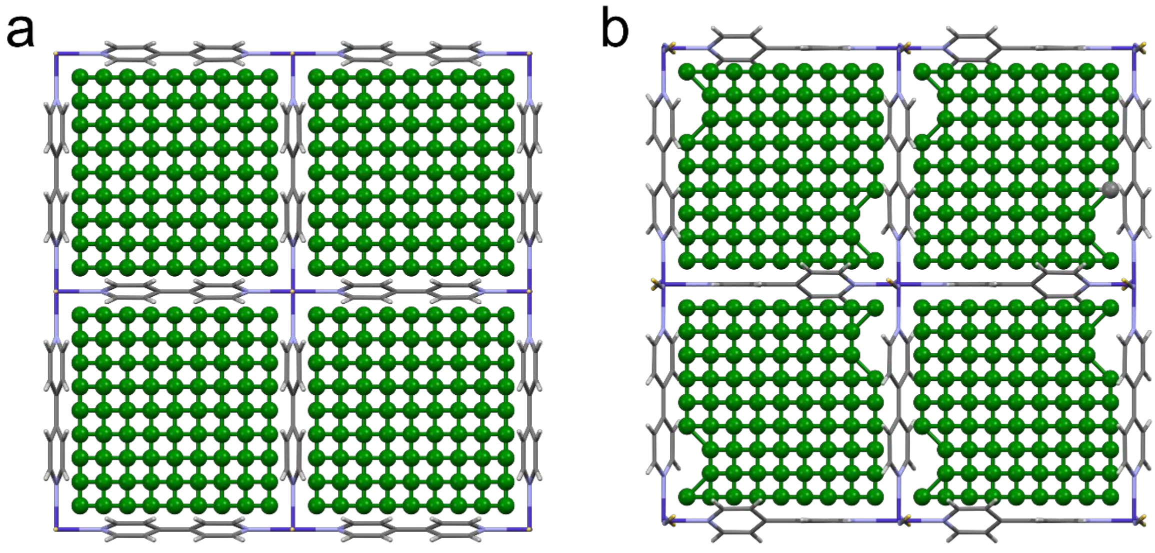

Figure S3. Fragment of a **sql** network in (a) sql-1-Co-NCS·4OX Phase I at 303 K/0.1 MPa and (b) Phase II at 100 K/0.1 MPa, shown along [001] and [100] directions, respectively. The dummy atoms inserted in the opening of the **sql** grid are shown in green as ball and stick. The dummy atoms were inserted in the same manner for all structures of sql-1-Co-NCS·4OX Phase I analyzed in this paper.

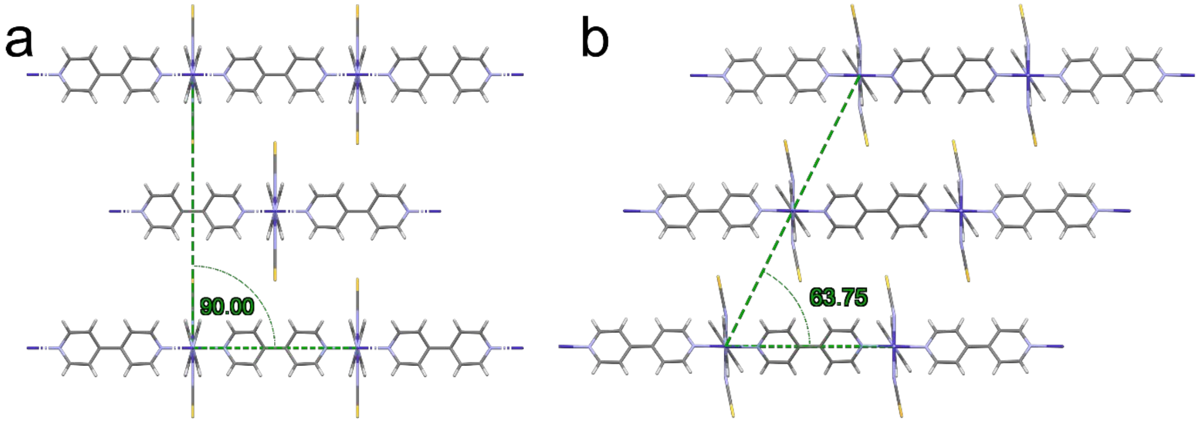

Figure S4. The crystal structure of sql-1-Co-NCS·4OX (a) Phase I at 303 K/0.1 MPa and (b) Phase II at 100 K/0.1 MPa (REFCODE KODDUF[1]) shown along directions [100] and [010], respectively. The angle between cobalt cations of translation-related **sql** networks is shown in green. For clarity for Phase II the OX molecules were omitted from the figure.

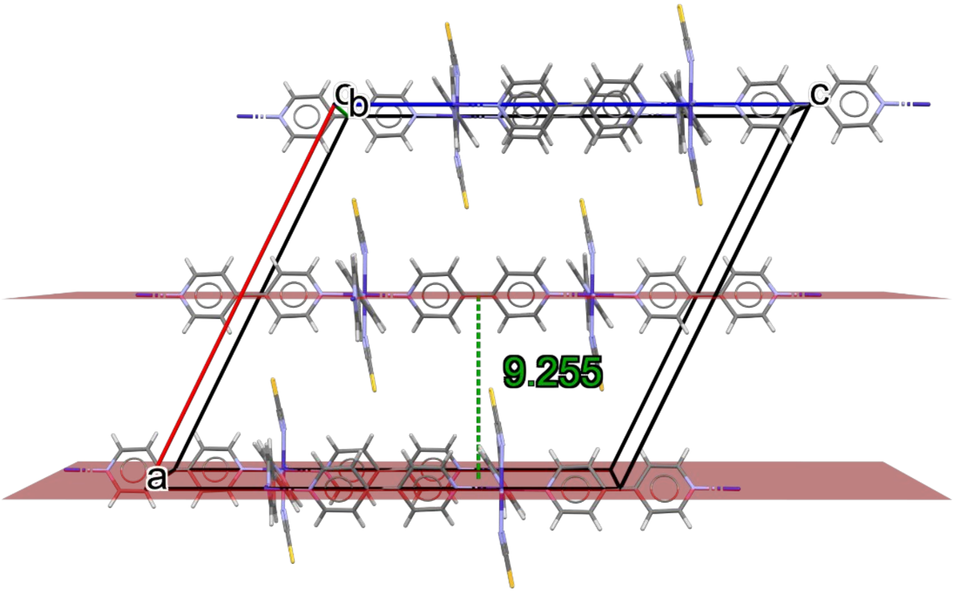

Figure S5. Figure showing the method for calculating the interlayer separation on an example of sql-1-Co-NCS·4OX Phase II at 100 K/0.1 MPa (REFCODE KODDUF[1]). The planes calculated for the Co cations of two **sql** layers are shown in red and interlayer separation is marked by green dashed line, with its value expressed in Å.

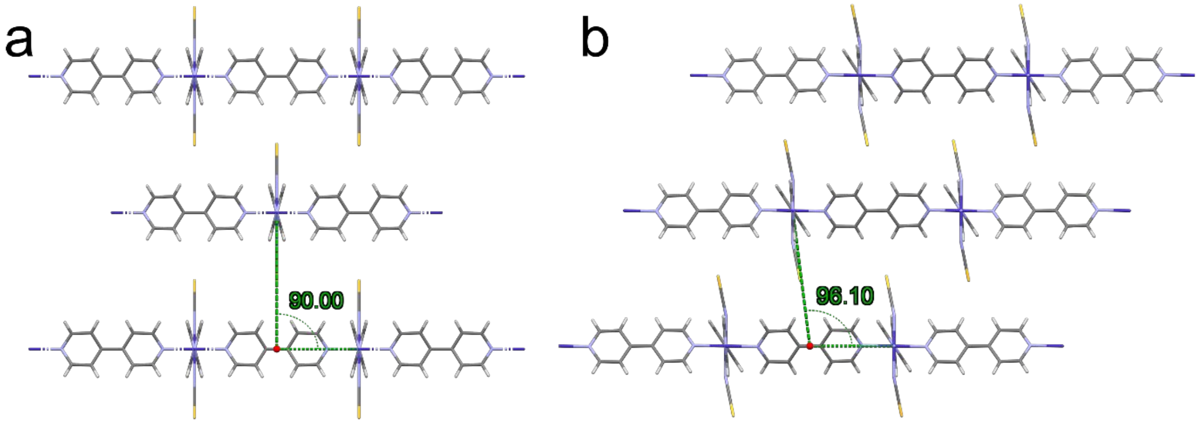

Figure S6. The crystal structure of sql-1-Co-NCS·4OX (a) Phase I at 303 K/0.1 MPa and (b) Phase II at 100 K/0.1 MPa (REFCODE KODDUF[1]) shown along directions [100] and [010], respectively. The Co-Centroid-Co angle between symmetry-related **sql** networks is shown in green. For clarity for Phase II the OX molecules were omitted from the figure.

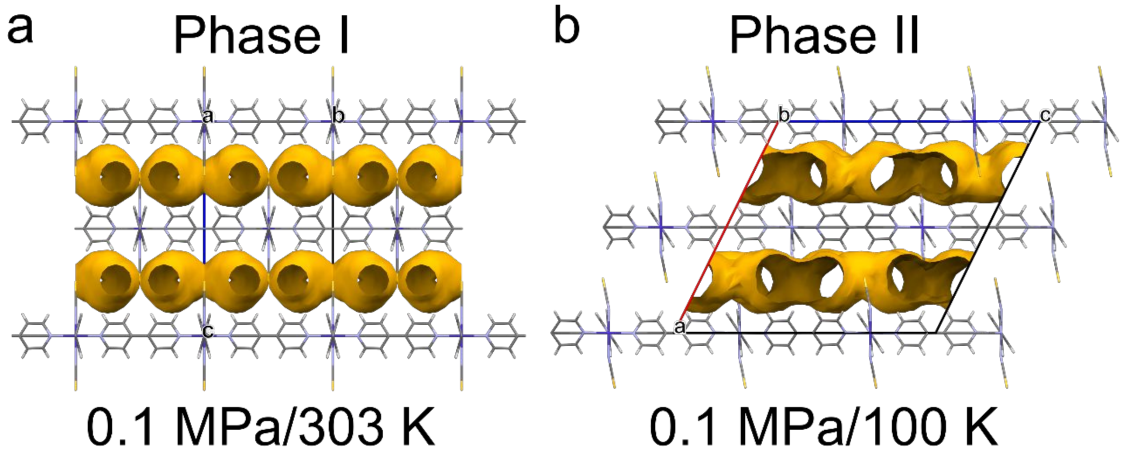

Figure S7. Structural voids (shown in orange) between the **sql** layers in structures of sql-1-Co-NCS·4OX (a) Phase I at 0.1 MPa/303 K shown along direction [100] and (b) Phase II at 0.1 MPa/100 K (REFCODE KODDUF[1]) shown along direction [010]. For structure of Phase II the OX molecules were removed from the structure. Voids were calculated for the contact surface, probe radius of 1.9 Å and grid spacing of 0.7 Å using program Mercury.[14]

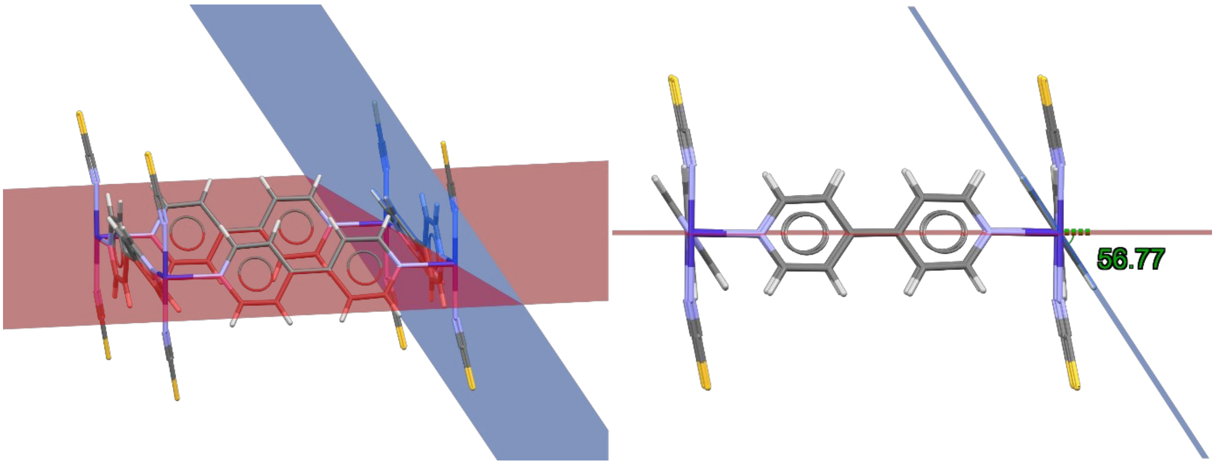

Figure S8. Figure showing the method for calculating the interlayer separation on an example of sql-1-Co-NCS·4OX Phase II at 100 K/0.1 MPa (REFCODE KODDUF[1]). The planes calculated for the Co cations of the **sql** layers and the selected pyridine ring of the 4,4'-bipyridine ligand, are shown in red and blue respectively. The angle between the planes (in [°]) is marked in green.

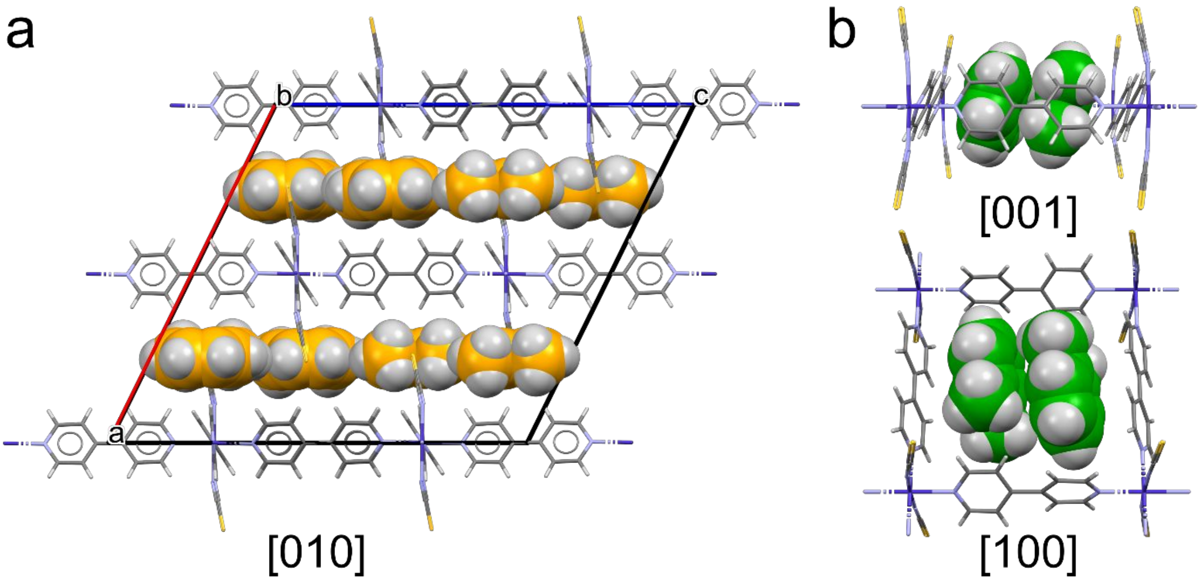

Figure S9. Molecular packing (a) and fragment of an **sql** network (b) in crystals of sql-1-Co-NCS·4OX Phase II at 100 K/0.1 MPa (REFCODE KODDUF[1]), shown along directions [010], and [001]/[100], respectively. The molecules of *o*-xylene are shown in spacefill style, with carbon atoms colored orange for molecules between **sql** layers, and green for molecules adsorbed in the grid cavity. For clarity of the figure, second components of the disordered molecules were omitted, and in section (a), *o*-xylene molecules placed within the **sql** grid were removed.

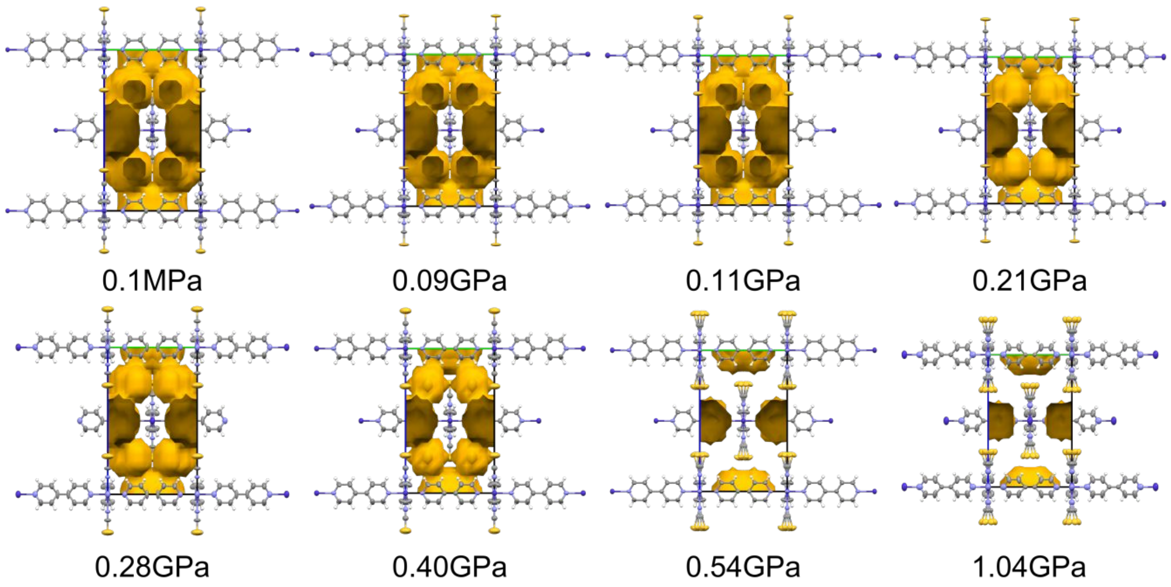

Figure S10. Structural voids (shown in orange) in structures of sql-1-Co-NCS·*x*OX Phases I, I*a* and I*b* (shown along direction [100]) in 0.1 MPa-1.04 GPa pressure range. Voids were calculated for the contact surface, probe radius of 1.9 Å and grid spacing of 0.7 Å using program Mercury.[14]

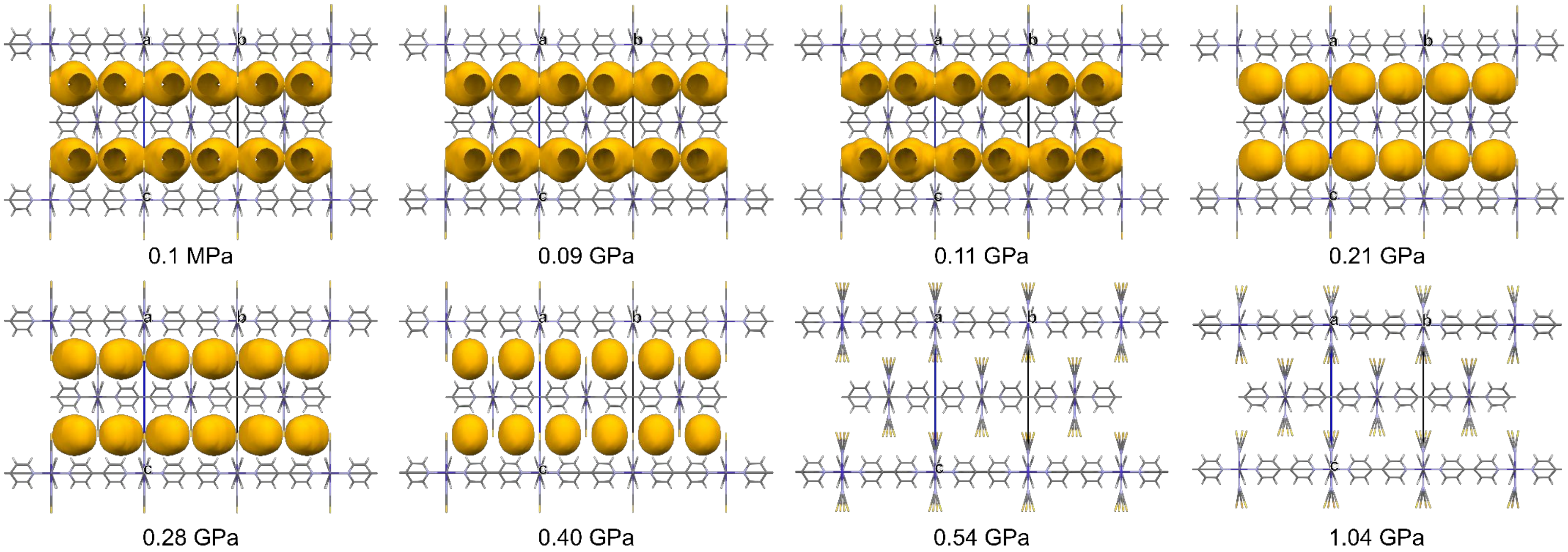

Figure S11. Structural voids (shown in orange) between the **sql** layers in structures of sql-1-Co-NCS·*x*OX Phases I, I*a* and I*b* (shown along direction [100]) in 0.1 MPa-1.04 GPa pressure range. Voids were calculated for the contact surface, probe radius of 1.9 Å and grid spacing of 0.7 Å using program Mercury.[14]

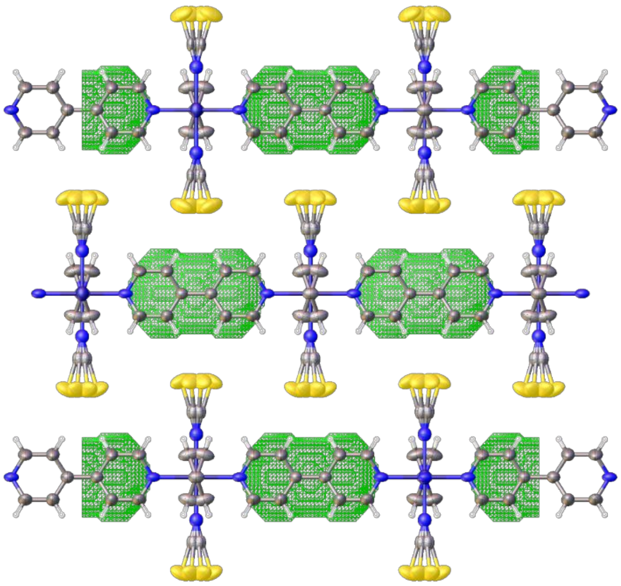

Figure S12. Structure of sql-1-Co-NCS·xOX at 298 K/0.54 GPa shown along direction [010] with masked volume shown in green. The mask was calculated with program Olex2.[15]

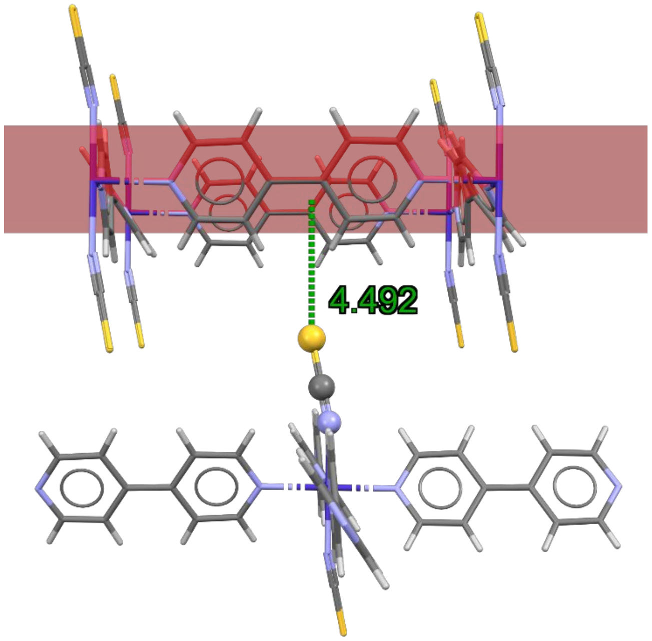

Figure S13. Fragments of two symmetry-related **sql** networks in crystals of sql-1-Co-NCS·4OX Phase II at 100 K/0.1 MPa (REFCODE KODDUF[1]), shown along direction [010], showing the way of measuring the distance between the sulfur atom of thiocyanate anion (shown in ball and stick style) and plane of the **sql** network (shown in red and calculated for four Co cations of the net) marked in green. The distance value is expressed in [Å]. The measurements were performed in analogous manner for all structures of sql-1-Co-NCS·4OX Phase I analyzed in this work. For clarity, the adsorbed OX molecules were removed from the figure.

3. References

(1) Wang, S.-Q.; Mukherjee, S.; Patyk-Kaźmierczak, E.; Darwish, S.; Bajpai, A.; Yang, Q.-Y.; Zaworotko, M. J. Highly Selective, High-Capacity Separation of o-Xylene from C8 Aromatics by a Switching Adsorbent Layered Material. *Angewandte Chemie International Edition* **2019**, *58* (20), 6630–6634. https://doi.org/10.1002/anie.201901198.

(2) Wriedt, M.; Näther, C. Preparation of New Ligand-Deficient Thiocyanato Compounds with Cooperative Magnetic Phenomena by Thermal Decomposition of Their Ligand-Rich Precursors. *European Journal of Inorganic Chemistry* **2010**, *2010* (20), 3201–3211. https://doi.org/10.1002/ejic.201000155.

(3) Wang, S.-Q.; Yang, Q.-Y.; Mukherjee, S.; O'Nolan, D.; Patyk-Kaźmierczak, E.; Chen, K.-J.; Shivanna, M.; Murray, C.; Tang, C. C.; Zaworotko, M. J. Recyclable Switching between Nonporous and Porous Phases of a Square Lattice (Sql) Topology Coordination Network. *Chem. Commun.* **2018**, *54* (51), 7042–7045. https://doi.org/10.1039/C8CC03838D.

(4) Lu, J.; Paliwala, T.; Lim, S. C.; Yu, C.; Niu, T.; Jacobson, A. J. Coordination Polymers of Co(NCS)2 with Pyrazine and 4,4'-Bipyridine: Syntheses and Structures. *Inorg. Chem.* **1997**, *36* (5), 923–929. https://doi.org/10.1021/ic961158g.

(5) Hao, M.-T.; Du, C.-F.; Tian, C.-B.; Li, J.-R.; Huang, X.-Y. [Fe(SCN)2(Bipy)2]·2(S8): A Two-Dimensional Coordination Polymer Intercalating the S8 Molecules. *Inorganic Chemistry Communications* **2016**, *72*, 128–131. https://doi.org/10.1016/j.inoche.2016.08.020.

(6) Wriedt, M.; Näther, C. Three New Iron(II) Thiocyanato Coordination Polymers Based on 4,4'-Bipyridine as Ligand and the Influence of Methanol on Their Structures. *Zeitschrift für anorganische und allgemeine Chemie* **2010**, *636* (6), 1061–1068. https://doi.org/10.1002/zaac.200900582.

(7) Adams, C. J.; Real, J. A.; Waddington, R. E. The Two-Dimensional Iron(II)–Thiocyanate–4,4'-Bipyridine Coordination Network. *CrystEngComm* **2010**, *12* (11), 3547–3553. https://doi.org/10.1039/C0CE00149J.

(8) Adams, C. J.; Muñoz, M. C.; Waddington, R. E.; Real, J. A. Cooperative Spin Transition in the Two-Dimensional Coordination Polymer [Fe(4,4'-Bipyridine)2(NCX)2]·4CHCl3 (X = S, Se). *Inorg. Chem.* **2011**, *50* (21), 10633–10642. https://doi.org/10.1021/ic200932w.

(9) Wang, S.-Q.; Darwish, S.; Sensharma, D.; Zaworotko, M. J. Tuning the Switching Pressure in Square Lattice Coordination Networks by Metal Cation Substitution. *Mater. Adv.* **2022**, *3* (2), 1240–1247. https://doi.org/10.1039/D1MA00785H.

(10) Zhang, Y.; Jianmin, L.; Wei, D.; Nishiura, M.; Imamoto, T. The Most Effective Packing of Layers: Synthesis and Structure of [Ni(4,4'-Bipyridine)2(NCS)2]n. *Chem. Lett.* **1999**, *28* (3), 195–196. https://doi.org/10.1246/cl.1999.195.

(11) Zhang, Y.; Jianmin, L.; Nishiura, M.; Imamoto, T. Spectral and Structural Properties of 2D Network Complex [Ni(4,4'–Bipyridine)2(NCS)2]n. *Journal of Molecular Structure* **2000**, *519* (1), 219–224. https://doi.org/10.1016/S0022-2860(99)00306-3.

(12) Wang, S.-Q.; Darwish, S.; Meng, X.-Q.; Chang, Z.; Bu, X.-H.; Zaworotko, M. J. Acetylene Storage Performance of [Ni(4,4'-Bipyridine)2(NCS)2]n, a Switching Square Lattice Coordination Network. *Chem. Commun.* **2022**, *58* (10), 1534–1537. https://doi.org/10.1039/D1CC06638B.

(13) Wriedt, M.; Jess, I.; Näther, C. Poly[[Bis-(μ-4,4'-Bipyridyl-K2N:N')Bis-(Thio-cyanato-KN)Manganese(II)] Diethyl Ether Disolvate]. *Acta Cryst E* **2010**, *66* (7), m781–m781. https://doi.org/10.1107/S1600536810021665.

(14) Macrae, C. F.; Sovago, I.; Cottrell, S. J.; Galek, P. T. A.; McCabe, P.; Pidcock, E.; Platings, M.; Shields, G. P.; Stevens, J. S.; Towler, M.; Wood, P. A. Mercury 4.0: From Visualization to Analysis, Design and Prediction. *J Appl Cryst* **2020**, *53* (1), 226–235. https://doi.org/10.1107/S1600576719014092.

(15) Dolomanov, O. V.; Bourhis, L. J.; Gildea, R. J.; Howard, J. A. K.; Puschmann, H. OLEX2: A Complete Structure Solution, Refinement and Analysis Program. *Journal of Applied Crystallography* **2009**, *42* (2), 339–341. https://doi.org/10.1107/S0021889808042726.
